# Hemorrhagic Tamponade as Initial Manifestation of Systemic Lupus with Subsequent Refractory and Progressive Lupus Myocarditis Resulting in Cardiomyopathy and Mitral Regurgitation

**DOI:** 10.1155/2018/7635982

**Published:** 2018-01-21

**Authors:** Nicole Marijanovich, Alexandra Halalau

**Affiliations:** ^1^Internal Medicine Department, Beaumont Hospital, Royal Oak, MI, USA; ^2^9 North Beaumont Hospital, Royal Oak, MI, USA; ^3^Oakland University William Beaumont School of Medicine, Rochester, MI, USA; ^4^General Internal Medicine Division, Beaumont Hospital, Royal Oak, MI, USA

## Abstract

Systemic lupus erythematosus (SLE) is a heterogeneous autoimmune disease with a wide range of clinical and serological manifestations. Cardiac disease among patients with SLE is common and can involve the pericardium, myocardium, valves, conduction system, and coronary arteries. We are reporting a case of SLE in a young woman that is unique is unique in that initial symptoms consisted of pericarditis and hemorrhagic tamponade which remained progressive and resistant to aggressive immunosuppressive treatment and led to severe cardiomyopathy (ejection fraction of 25%) and severe (+4) mitral regurgitation. Her immunosuppressive treatment included hydroxychloroquine, high-dose steroids, intravenous immunoglobulins, azathioprine, and mycophenolate mofetil. Her disease progression was felt to be due to underlying uncontrolled SLE because the complement levels remained persistently low throughout the entire course and PET Myocardial Perfusion and Viability study showed stable persistent active inflammation. Eventually, she was treated with cyclophosphamide which led to improvement in ejection fraction to 55% with only mild mitral regurgitation.

## 1. Introduction

Systemic lupus erythematosus (SLE) is an autoimmune condition that can be associated with significant morbidity and mortality. Pericarditis is the most common cardiac manifestation of SLE; however, pericardial effusions causing pericardial tamponade are rare. There is a 1–2.5% incidence of tamponade in patients with systemic lupus erythematosus [1]. Myocarditis is clinically prevalent in 9% of those who have SLE [2]. Along with congestive heart failure, myocarditis contributes approximately 31% to cardiac deaths in inflammatory myopathies and indicates a poor prognosis [3]. Therefore, prompt recognition, diagnosis, and treatment of pericardial tamponade and myocarditis are imperative.

## 2. Case Presentation

A 21-year-old female of mixed heritage (Caucasian and African American), with a history of provoked deep venous thrombosis diagnosed six months before, currently treated with warfarin, presented with complaints of acute chest pain. The pain was midsternal, severe, sharp, and worse with inspiration but improved by sitting upright. Vital signs revealed tachycardia (154 beats per minute), tachypnea (30 breaths per minute), and normal blood pressure (136/73 mmHg). The INR was 5.4 on admission. The chest X-ray showed cardiomegaly with a bottle neck sign. The echocardiogram showed a large pericardial effusion, with evidence of right-sided collapse during diastole. Chest, abdomen, and pelvis CT demonstrated a large pericardial effusion, consistent with hemorrhage. There were no pulmonary emboli or signs of pulmonary hemorrhage. The patient was transfused plasma and blood with improvement of the INR to 2.1. Pericardiocentesis was performed with a return of 500 cc of bloody fluid, and a drain was placed. Her vital signs improved and symptoms diminished immediately after the procedure.

She was diagnosed with underlying pericarditis with effusion that converted to a hemorrhage from the anticoagulation. The etiology of her pericarditis was sought. She denied any recent viral illnesses, tuberculosis exposure, malignancy, or fungal infections. She denied any arthritis, oral ulcers, fever, weight loss, night sweats, rash, or family history of autoimmune disease. Pericardial fluid was sent for cytology as well as viral, fungal, aerobic, anaerobic, and AFB cultures that were all negative. No autoimmune testing was performed on the pericardial fluid. Initial laboratory work revealed normal electrolyte panel and renal function and BNP 59 pg/ml (0–100). She had mildly elevated liver function tests with AST 69 U/L (10–37) and ALT 52 U/L (8–37). She was also found to have normocytic anemia. The hemoglobin was 9 g/dl (baseline was 11.1) with a normal white blood cell and platelet count. Inflammatory markers were elevated: erythrocyte sedimentation rate was 35 mm/hr (0–18), C-reactive protein was 10.4 mg/dl (0–0.8), and ferritin was 314 ng/ml (12–207).

Autoimmune evaluation showed an ANA titer of 1 : 1280 with speckled pattern and markedly elevated anti-double-stranded DNA 195 IU/ml (<100). SSA Ab and SSB Ab were also elevated at 187 and 538 AU/ml (<100), respectively. The patient did not have any clinical symptoms to suggest sicca syndrome (no dry eyes or mouth). TSH on admission was 0.60 (0.5–5.20 mcIU/ml) with repeat serial TSH remaining in normal limits. Clinically, she was never symptomatic with thyroiditis. Complement C4 was low at 7 mg/dl (12–43). Rheumatoid factor was 27 IU/ml (0–14). ANCA, SPEP, CCP, anti-Smith Ab, RNP Ab, and complement C3 were normal. Initial antiphospholipid screen (APS) was positive; however, repeat testing approximately 12 weeks later was negative. Initial APS enzyme-linked immunosorbent assay (ELISA) showed anti-cardiolipin Ab IgA < 9.5 (<12 APL), IgG 21.6 (<15 GPL), IgM 19.7 (<12.5 MPL), beta-2 glycoprotein 1 IgG 12.1 (<20 SUG), and beta-2 glycoprotein I IgM 24.5 (<20 SMU). Repeat APS panel was negative with all results at <9.5. DRVVT screen was 50 (0–52 seconds). The patient was determined to not have antiphospholipid syndrome due to lack of positive repeat APS Ab blood work. Infectious work-up including VDRL, HIV, pericardial fluid Gram-stain, culture, AFB stain and culture, and virus culture was negative. Pericardial fluid cytology was negative for malignancy and revealed increased neutrophils and lymphocytes.

The patient was diagnosed with systemic lupus erythematosus and was started on intravenous (IV) methylprednisolone 100 mg for 3 days with transition to prednisone 40 mg daily, hydroxychloroquine 200 mg twice daily, and colchicine 0.6 mg twice daily. She was also treated with ibuprofen 400 mg every 6 hours and opioids for pain control. Over the next 48 hours, an additional 120 cc bloody fluid was drained after repositioning the pericardial drain, which was then successfully removed. In hospital day 3, troponin peaked at 3.09 ng/dl (0–0.05) and lupus myocarditis was considered. A cardiac MRI showed normal LV function and no evidence of myocarditis. However, it did reveal severe diffuse, circumferential pericardial contrast uptake involving both the visceral and parietal pericardium with trivial pericardial effusion. She continued to improve and was discharged after 8 days from the hospital with colchicine 0.6 mg twice daily, hydroxychloroquine 200 mg daily, and prednisone 40 mg daily. Colchicine was used to treat the patient for inflammatory pericarditis as imaging revealed inflammation, and she remained symptomatic despite evacuation of pericardial fluid.

Three weeks later, she presented with recurrent chest pain despite compliance with her medications. ECG showed new T-wave inversions in multiple leads and troponin peaked at 4 ng/dl (0–0.05). BNP was elevated at 742 pg/ml (0–100). A transesophageal echocardiogram showed an ejection fraction of 45% with mild-moderate mitral regurgitation. A cardiac MRI showed midmyocardial and epicardial hyperenhancement in the midlateral and inferolateral walls, suggestive of myocarditis. It also revealed persistent pericarditis similar to prior cardiac MRI but no pericardial effusion or constriction. Myocardial perfusion imaging showed fixed radiotracer defect in the mid-distal inferior and inferolateral walls concerning for myocardial scar. PET Myocardial Perfusion and Viability study showed resting hypoperfusion in the midinferior lateral region, which was matched on FDG suspected to be scar formation and flow-metabolic mismatch in the basal inferior and basal inferolateral regions, approximating 12% of the left ventricle. The myocardial scar was estimated to be 6% of the left ventricle myocardium. She was started on aspirin and carvedilol 3.125 mg twice daily. She was again treated with IV steroids and started on azathioprine 50 mg twice daily. Repeat anti-double-stranded DNA had decreased to 130 IU/ml (<100); however, complements C3 and C4 remained low at 76 mg/dl (80–200) and <6 mg/dl (12–43), respectively. Her symptoms improved, and she was discharged home after 6 days from the hospital.

Six weeks later, she presented to the hospital again with recurring chest pain that was associated with bilateral leg swelling, dyspnea with exertion, and orthopnea. BNP was markedly elevated at 1098 pg/ml (0–100). Transesophageal echocardiogram also revealed EF 35% with left ventricular global hypokinesis and moderate-severe mitral regurgitation. Infectious endocarditis was ruled out with serial blood cultures, and no vegetations were found on imaging. A third cardiac MRI was performed which showed a severely depressed ejection fraction 29% and midinferolateral wall akinesis with full thickness hyperenhancement. When compared to the prior MRI, it also showed progression of her myopericarditis.

Cardiac catheterization showed normal coronary arteries, ejection fraction 25%, global hypokinesis, and severe +4 mitral regurgitation with severely enlarged left atrium. Cardiovascular surgery was consulted regarding evaluation for mitral valve repair; however, recommendations were made to pursue surgery only if she were to fail maximum medical treatment. Given no recommendations for surgery, invasive myocardial biopsy was deferred as well. She was treated for acute decompensated congestive heart failure with IV furosemide, and later adequate diuresis was transitioned to oral furosemide 20 mg daily. She was also started on spironolactone 25 mg daily, carvedilol 6.25 mg twice daily, and lisinopril 5 mg daily.

Interestingly, a repeat anti-double-stranded DNA was normal at 83 IU/ml (<100); however, complement C4 remained low at 6 mg/dl (12–43). Given progression of her disease despite medication compliance, she was treated with 1 gram IV methylprednisolone for 3 days along with IVIG for 2 days. She was continued on hydroxychloroquine sulfate 200 mg twice daily and colchicine. Azathioprine was eventually discontinued and changed to mycophenolate mofetil 500 mg twice daily with plans to increase to 1000 mg twice daily.

She was monitored by serial echocardiograms which showed stabilization and mild improvement in her valve function. She was then discharged home after 12 days from the hospital. Repeat PET Myocardial Perfusion and Viability study performed 3 weeks later (and 4 months after initial presentation) showed similar findings to a prior study with active inflammation of the basal inferior, and basal inferior lateral regions were estimated to be 12% of the left ventricle myocardium. Myocardial scar/fibrosis was estimated to be 8% of the left ventricle myocardium. She was then started on cyclophosphamide with repeat echocardiogram showing improvement in ejection fraction to 36% and mild-moderate mitral regurgitation and complement levels improving. Fortunately, her most recent echocardiogram (2.5 years after initial diagnosis) reveals an EF of 55%, mild mitral regurgitation, and no hypokinesis appreciated. Her complement levels and anti-double-stranded DNA remain within normal range and no other manifestations of SLE. Unfortunately, due to prolonged, high-dose steroid use for over 1 year, she developed avascular necrosis of her bilateral femoral heads and condyles necessitating surgery on her bilateral knees and hip. Currently, her only immunosuppressive medication remains hydroxychloroquine.

## 3. Discussion

Our patient was very unusual because cardiac tamponade as a result of lupus-induced pericarditis was her initial presenting symptom of lupus and because her disease remained refractory and even progressed to myocarditis, resulting in severe mitral regurgitation and cardiomyopathy. In one retrospective study and literature review, 41 patients (out of 71 patients with SLE) were found to have tamponade. Patients with pericardial effusions who developed tamponade had a statistically significant (*P* < 0.05) lower C4 level at presentation as compared with patients who did not develop tamponade. This is consistent with our patient, who despite normalization of her anti-double-stranded DNA, continued to have consistently low C4 levels during her active disease. Decreased serum C4 levels, female sex, concurrent renal disease, haemolytic anemia, and pleurisy may all be potential predictors of tamponade in SLE patients with pericarditis and effusion [1].

Myocarditis has a varied clinical manifestation and can present with dyspnea, fever, orthopnea, chest pain, peripheral edema, or palpitations. The gold standard for confirmation of myocarditis is endomyocardial biopsy. However, the focal nature of myocarditis leads to significant sampling error with biopsy and, consequently, low sensitivity while also exposing the patient to the risk of complications including cardiac perforation and stroke [2]. In recent years, clinically overt myocarditis is uncommon, reported in only 7–10% of cases, probably as the consequence of the introduction of steroid therapy. Cardiac histological findings show small foci of fibrinoid necrosis with infiltrates of plasma cells and lymphocytes and small foci of myocardial fibrosis. Immunofluorescence studies demonstrate fine granular immune complexes and complement deposition in the walls and perivascular tissues of myocardial blood vessels, supporting the hypothesis that lupus myocarditis is an immune complex-mediated vascular phenomenon [3].

In one case series of lupus myocarditis, which looked at 24 patients, a bimodal age distribution was found. The data suggested that although patients with newly diagnosed SLE and lupus myocarditis are acutely ill at presentation, their prognosis is good with proper treatment. It also found that low left ventricular ejection fraction on presentation, which does not improve with treatment, is an indicator of poor prognosis. Eleven of the 22 patients had considerable valvular dysfunction on the initial echocardiogram with most (81%) having mitral regurgitation. The most likely cause in the majority of patients seems to be a functional change in the ventricular geometry, resulting in annular dilation [2] as was seen in our patient. In those patients who did not recover their systolic function, the valvular dysfunction persisted. Echocardiography was a useful investigation in monitoring the response to the therapy; however, because of the nonspecific findings, cardiac MRI is the diagnostic method of choice. Along with the aforementioned treatment recommendations, standard-of-care pharmacotherapy of patients with congestive heart failure is also recommended [2].

Monitoring active SLE disease is difficult and limited. Currently, there are scoring tools such as the British Isles Lupus Assessment Group (BILAG) index, the Systemic Lupus Activity Measure (SLAM) index, and Safety of Estrogens in Lupus National Assessment study-SLEDAI that attempt to measure disease activity and guide physicians on when to escalate treatment. However, these metrics often have administrative burden as well as other significant limitations. Traditional serum biomarkers can often reveal active disease and can help predict organ involvement. Unfortunately, they are not always accurate, and there are no clinical trials to date supporting routine use for monitoring disease activity. Therefore, in order to monitor active disease in our patient, we used a combination of clinical assessment, serum biomarkers, and imaging. A new promising lupus biomarker is the interferon signature characterized by the highly coordinated upregulation of type I IFN-inducible inflammatory cytokines, chemokines, and other genes whose expression levels are closely correlated with clinical and laboratory measures of SLE disease activity. This can lead to identification of patients at high risk for specific organ involvement and personalized, targeted treatment [4].

There are no standard guidelines for treating lupus myocarditis; most case reports have demonstrated a favorable response to pulse corticosteroids or cyclophosphamide along with supportive management [5]. IVIG is being increasingly used for the treatment of autoimmune diseases, including SLE, in an attempt to control severe manifestations unresponsive to other treatments. Perhaps, the most dramatic case report was a 20-year-old female with recent diagnosis of lupus (with active myositis treated with oral prednisone and azathioprine) who presented with acute myocarditis as evidenced by an ejection fraction of 15% and large pericardial effusion. Left and right cardiac catheterization was unremarkable, but she required intra-aortic balloon pump and soon developed cardiogenic shock. She subsequently arrested and received a pericardial window with biventricular assist devices. She was started on stress-dose steroids and IVIG with marked improvement, and within a week, her biventricular devices were removed. Within a month, her EF and creatine kinase had normalized [3].

There are no prospective controlled trials of IVIG in SLE, but many published case series have shown improvement of cutaneous, musculoskeletal, neuropsychiatric, serositic, haematological, vasculitic, and nephritic manifestations of SLE. There are few reports of lupus myocarditis treated with IVIG, but all have shown rapid improvement in the clinical and echocardiographic abnormalities [6]. The duration for treatment with IVIG is undetermined with ranges from 2 to 5 days. The standard dose used is 1 g/kg in most case reports. Although our patient did not show a significant improvement in her ejection fraction immediately, she clinically improved after treatment with IVIG and her active inflammation seemed to dissipate as evidenced by normalization of anti-dsDNA antibody levels and stabilization in her echocardiogram and valve dysfunction.

In more severe cases or in cardiac tamponade, a higher dose of corticosteroid is necessary, often given as an intravenous bolus (such as 1 gram of methylprednisolone daily for 3 days). In patients with recurrent pericarditis, chronic immunosuppression with methotrexate, azathioprine, or mycophenolate mofetil and the use of intravenous immunoglobulin may be beneficial [7]. In our case, the patient's underlying disease slowly progressed while on azathioprine. The overall disease progression was halted on mycophenolate mofetil, but the active inflammation was persistent in the myocardium. Only after receiving the cyclophosphamide therapy, the patient's underlying myocardium inflammation resolved and led to improvement in her ejection fraction and degree of mitral regurgitation.

In summary, while cardiac treatment modalities in SLE remain a challenge, patients who present with severe, life-threatening cardiac involvement justify the use of earlier and more aggressive immune therapy in order to achieve and maintain resolution of disease and improvement in ejection fraction.
